# Recurrent cellulitis associated with lymphoedema in Noonan syndrome: case reports with *RIT1* variants and literature review

**DOI:** 10.1038/s41439-025-00315-1

**Published:** 2025-06-04

**Authors:** Yuki Kobayashi, Takeya Adachi, Umi Tahara, Moemi Tanaka, Hiroki Arakawa, Yohei Funatsu, Kazunori Moritani, Mamiko Yamada, Kenjiro Kosaki, Toyoko Inazumi

**Affiliations:** 1 Department of Dermatology, Tachikawa Hospital, Federation of National Public Service Personnel Mutual Aid Associations, Tokyo, Japan; 2 Department of Dermatology, Keio University School of Medicine, Tokyo, Japan; 3 Department of Medical Innovation and Translational Medical Science, Graduate School of Medical Science, Kyoto Prefectural University of Medicine, Kyoto, Japan; 4 Department of Infectious Control Team, Tachikawa Hospital, Federation of National Public Service Personnel Mutual Aid Associations, Tokyo, Japan; 5 Department of Cardiology, Tachikawa Hospital, Federation of National Public Service Personnel Mutual Aid Associations, Tokyo, Japan; 6 Center for Medical Genetics, Keio University School of Medicine, Tokyo, Japan

**Keywords:** Disease genetics, Genetic association study

## Abstract

Noonan syndrome (NS) is a RASopathy, a disorder caused by genetic alterations involving the Ras/mitogen-activated protein kinase pathway. It causes characteristic clinical manifestations, including facial dysmorphism and congenital cardiac defects. Occasionally, lymphoedema and recurrent cellulitis occur in patients with NS, potentially escalating to lethal conditions. Despite the frequent association of cellulitis with lymphoedema in NS, features susceptible to these complications have not been fully characterized. We encountered two patients with NS carrying *RIT1* pathogenic variants, who were treated for recurrent lower leg cellulitis since their teenage years, which occasionally progressed to sepsis. Here we retrospectively examined these patients with NS and recurrent cellulitis on the background of lymphoedema and reviewed published cases of NS with lymphoedema and cellulitis up to March 2024 to elucidate the clinical and genetic features of this subgroup. Our literature review identified 16 additional patients with NS with similar complications. Among the 18 patients (15 men), genetic analyses revealed pathogenic variants in *PTPN11* and *RIT1* in 4 patients each, with the latter occurring more frequently than commonly observed. The patients developed lymphoedema by 15 years of age, predisposing them to cellulitis by 23 years of age. Notably, four of the five patients with sepsis had congenital heart defects, with a higher prevalence than that generally reported in NS. This study highlights the characteristics of genetic variants, congenital cardiac anomalies and heightened risk of recurrent cellulitis in patients with NS, emphasizing the need for early intervention with prophylactic antibiotics and surgical treatment to mitigate these risks.

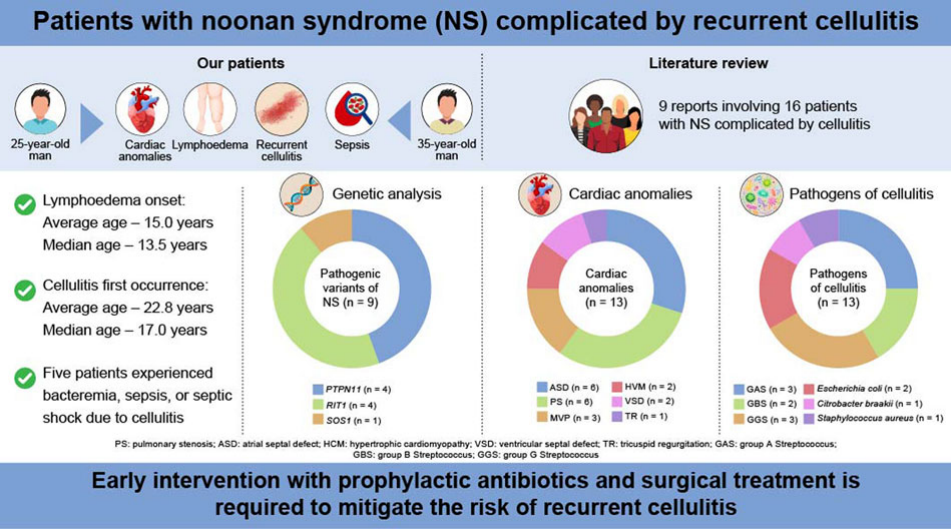

## Noonan syndrome linked to increased cellulitis and lymphoedema risks

Noonan syndrome (NS) is a genetic disorder that affects various parts of the body, often leading to heart defects and unique facial features. Researchers have identified several genes linked to NS, but the connection between these genes and certain complications, such as cellulitis and lymphoedema, is not well understood. In this study, two patients with NS and recurrent cellulitis were examined. Researchers collected clinical data from these patients and conducted genetic testing to identify any mutations. They also reviewed 15 similar cases from existing literature. The study found that both patients had a mutation in the *RIT1* gene, which is known to be associated with NS. This mutation may increase the risk of developing lymphoedema and cellulitis. The findings suggest that specific genetic mutations in patients with NS could lead to recurrent infections and swelling.

This summary was initially drafted using artificial intelligence, then revised and fact-checked by the author.

## Introduction

Noonan syndrome (NS) is a RASopathy, a clinically diverse disorder caused by germline alterations in the genes involved in the Ras/mitogen-activated protein kinase pathway^1^. Several causative genes for NS, including *PTPN11*, *SOS1*, *SOS2*, *RIT1*, *RAF1*, *KRAS*, *NRAS*, *BRAF* and *LZTR1*, have been identified^2,3^. Most NS cases follow an autosomal dominant inheritance pattern, except for autosomal recessive forms of *LZTR1*-associated NS^4^.

NS was first identified by Noonan and Ehmke in 1963, with distinctive traits, including low-set posteriorly rotated ears, hypertelorism, ptosis, short stature and congenital heart defects^5^. Lymphatic anomalies, including lower limb lymphoedema, genital swelling and systemic manifestations, such as intestinal lymphangiectasia and chylothorax, are other NS complications^6^. Diagnostic imaging has revealed morphological and functional lymphatic system abnormalities, including systemic lymphatic hyperplasia and impaired transport on magnetic resonance lymphangiography^7^, alongside venous incompetence on duplex scans^6^. Lymphoedema is a predisposing factor for cellulitis, which can escalate to bacteremia, sepsis or septic shock, posing life-threatening risks^8^. Moreover, patients with NS may develop elephantiasis owing to recurrent cellulitis of the lower limbs, substantially impacting their quality of life. Despite the frequent association of cellulitis with lymphoedema in NS, susceptibility features to these complications have not been fully characterized.

This Article details our encounter with two patients with NS harboring pathogenic variants in *RIT1*. Both patients experienced recurrent cellulitis and lymphoedema. We aimed to provide insights into this by reporting our findings and conducting a review of 15 similar cases from the literature, focusing on their clinical and genetic features.

## Methods

### Patients and sample collection

We enrolled two patients with NS and cellulitis at Tachikawa Hospital, Federation of National Public Service Personnel Mutual Aid Associations. We retrospectively analyzed their clinical data collected from patient records, including age, sex, laboratory findings, treatments and outcomes. The Ethics Committee of Tachikawa Hospital approved this study (approval number 2021-13), and written informed consent for publication, including images, was obtained from both patients.

### Genetic testing

Peripheral blood samples were collected from patients after obtaining informed consent. Genetic analysis was conducted through next-generation sequencing at the Kazusa DNA Research Institute, using a custom panel from Twist Biosciences as a hybrid capture probe, as previously described^9^. The covered genes in the diagnostic gene panel test for patients with suspected NS were *PTPN11*, *SOS1*, *RAF1*, *RIT1*, *NRAS*, *BRAF*, *SHOC2*, *CBL*, *BRAF*, *HRAS*, *MAP2K1* and *MAP2K2*.

### Bacterial testing

Two sets of aerobic and anaerobic blood cultures were collected upon admission, and susceptibility tests were performed. After an initial positive blood culture result, follow-up blood cultures were obtained repeatedly every 3–4 days until the results were negative.

### Literature review

#### Search strategy and selection criteria

**Fig. 1:**
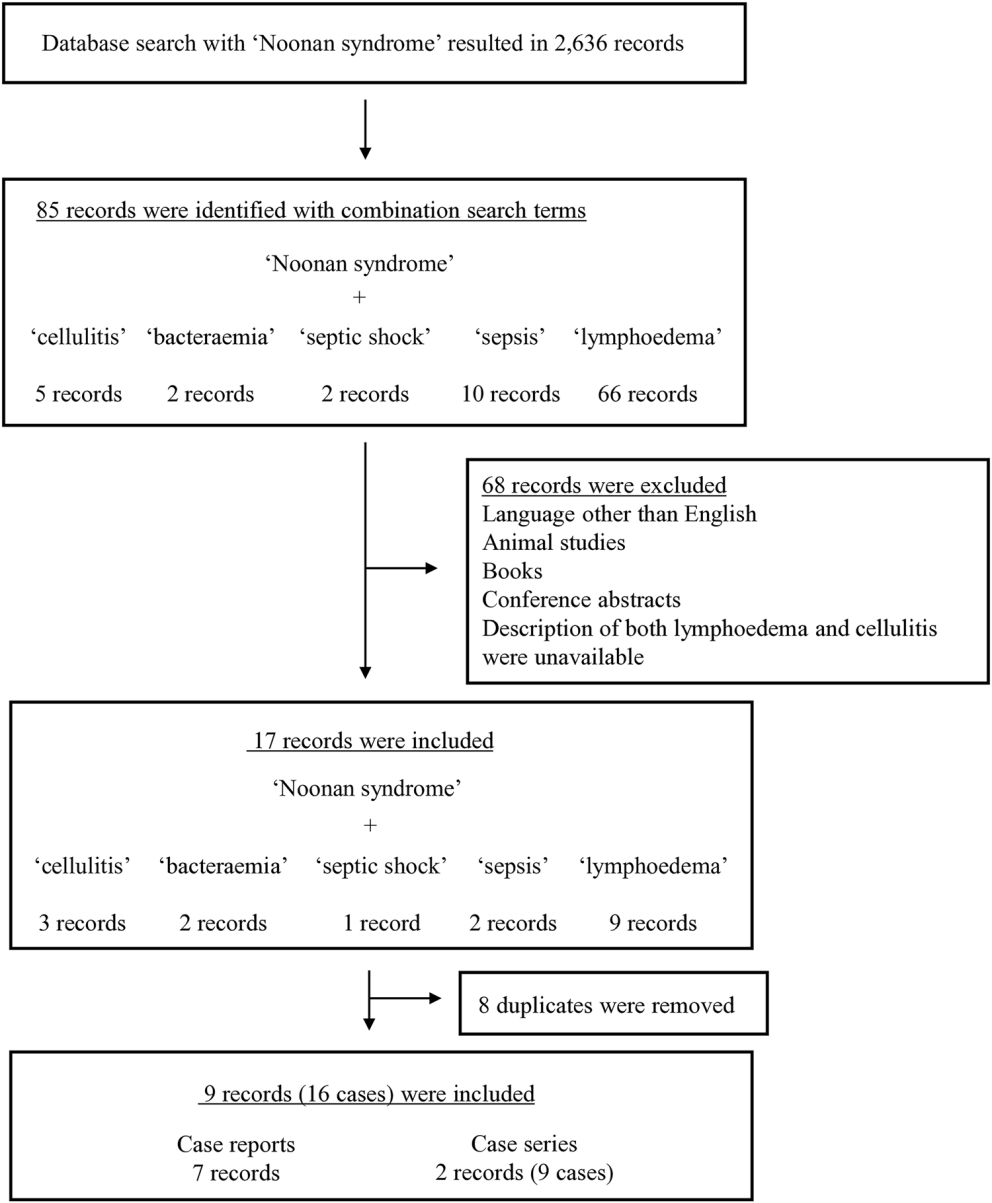
Flow diagram outlining study inclusion for NS complicated with lymphoedema and cellulitis. The literature search across PubMed with the MeSH term ‘Noonan syndrome’ combined with text words such as ‘cellulitis’, ‘bacteraemia’, ‘septic shock’, ‘sepsis’ or ‘lymphoedema’ resulted in 85 hits. Based on the title, abstract and full text, 68 records were excluded because they did not meet the eligibility criteria or were not available in full text, and 8 duplicates were removed. In total, 9 articles (7 case reports and 2 case series) were identified, involving 16 patients with NS complicated by lymphoedema and cellulitis.

An electronic search in PubMed was conducted to retrieve all publications on studies about NS and cellulitis. To identify relevant studies, selected medical subject heading (MeSH) terms and text words were used. The MeSH term ‘Noonan syndrome’ was combined with text words, such as ‘cellulitis’, ‘bacteraemia’, ‘septic shock’, ‘sepsis’ or ‘lymphoedema’ (Fig. 1).

Studies on patients diagnosed with NS and in whom lymphoedema and cellulitis were reported and those published before 1 March 2024 were included. Studies published in a language other than English, animal studies, books, conference abstracts and reports in which descriptions of both lymphoedema and cellulitis were unavailable were excluded. To assess whether the inclusion criteria were met, all articles were screened by their title and abstract, followed by full-text screening by two investigators (Y.K. and M.T.).

#### Data extraction and descriptive analysis

Results from case series were extracted from the text and tables. Results from case reports were extracted from the text. The genetic findings and clinical features, including age, sex, cardiac anomalies, lymphoedema onset age, cellulitis onset age, cellulitis frequency, causative bacteria, sepsis history and treatment approaches, were extracted and analyzed whenever possible. Data extraction and analysis were performed by two authors (Y.K. and M.T.). Disagreements concerning the extracted data were resolved through discussion with a third reviewer (T.A.).

## Results

### Case reports

#### Patient 1

A 25-year-old man with a history of NS diagnosed in infancy was admitted to our department owing to lower limb swelling, a high body temperature of 40.5 °C, fatigue, headache, arthritis and abdominal pain, prompting diagnoses of cellulitis and sepsis. His medical history was notable for cardiac abnormalities, including pulmonary stenosis (PS), atrial septal defect (ASD) and hypertrophic cardiomyopathy (HCM), along with bilateral cryptorchidism, scrotal lymphorrhoea and lymphoedema of both lower extremities, manifesting at age 14. He had experienced 11 episodes of cellulitis requiring hospitalization since age 16, one of which escalated to septic shock, disseminated intravascular coagulation and acute respiratory distress syndrome at the age of 19 years. His surgical history included ASD repair, four laparoscopic surgeries for cryptorchidism, lymphovenous anastomosis at the age of 17 years, scrotal lymphatic resection at the age of 23 years and excessive skin resection of the penis at the age of 24 years. The patient was monitored biannually for ASD.

**Fig. 2:**
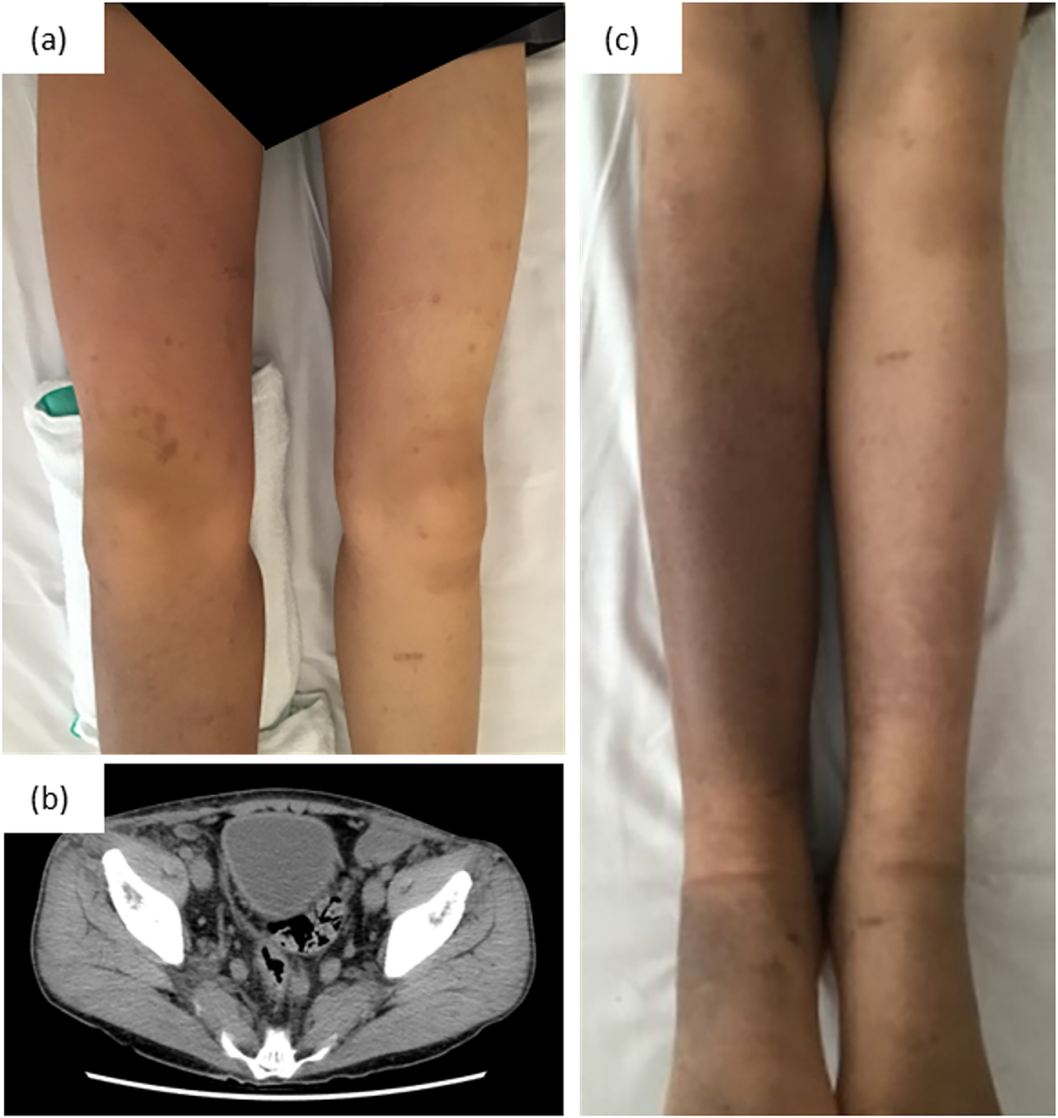
Clinical features of patient 1. **a** Physical examination reveals bilateral lower limb swelling, more severe in the right leg, with accompanying redness extending to the right inguinal region. **b** Computed tomography showing multiple enlarged pelvic lymph nodes. **c** Swelling and redness improved after antibacterial treatment.

Upon admission, a physical examination revealed bilateral lower limb swelling, which was more severe in the right leg, with accompanying redness and pain extending to the right inguinal region and abdomen (Fig. 2a). Laboratory tests revealed elevated C-reactive protein and procalcitonin levels. Transthoracic echocardiography did not reveal vegetation-like masses. Computed tomography revealed multiple enlarged pelvic lymph nodes (Fig. 2b). Given his history of recurrent cellulitis, treatment was commenced with ceftriaxone 2 g per day to cover potentially resistant bacteria. Subsequently, blood cultures revealed *Streptococcus pyogenes* (Group A *Streptococcus* (GAS)), prompting cefazolin administration (3 g per day). His symptoms improved (Fig. 2c), and the blood culture result returned negative (day 6), demonstrating an effective response to the adjusted antibiotic regimen; he was discharged on day 13. Cellulitis recurred twice within 1 year even after a sufficient amount and duration of antibiotic therapy. Genetic testing revealed a heterozygous variant, *RIT1*(OMIM, Online Mendelian Inheritance in Man: 609591) chr1(GRCh37:g.155874285A>T), NM_006912.6: c.246T>A (p.Phe82Leu), that was previously reported as a pathogenic variant^10^.

#### Patient 2

**Fig. 3:**
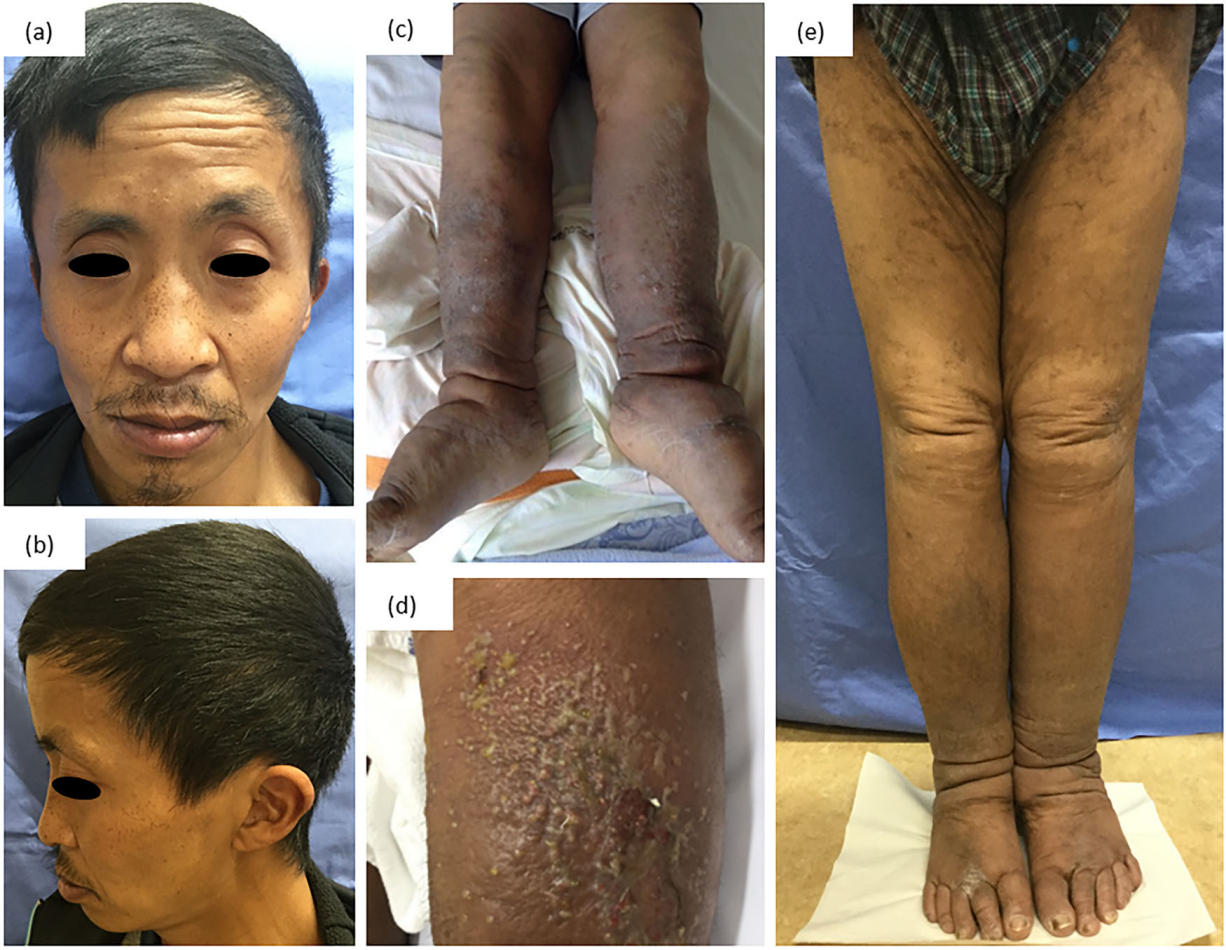
Clinical features of patient 2. **a**, **b** Facial dysmorphism, including ptosis and low-set posteriorly rotated ears (**a** Frontal view **b** Lateral view). **c**, **d** Physical examination reveals bilateral limb lymphoedema, more severe in the left leg (**c**), accompanied by exudate (**d**). **e** Lymphoedema improved after vascularized lymph node transplantation, and no further recurrence was observed for 3 years.

A 35-year-old man was diagnosed with NS in early childhood on the basis of clinical features, including facial dysmorphism (Fig. 3a, b), cardiac anomalies, lymphatic dysfunction and mild intellectual disabilities. His complex medical history included a diaphragmatic hernia, cardiac abnormalities including mitral valve prolapse and tricuspid regurgitation, mild intellectual disabilities, bilateral lower limb lymphoedema and recurrent cellulitis episodes starting from adolescence. In addition, he experienced macroscopic hematuria and chyluria, intestinal obstruction and a critical episode of septic shock with disseminated intravascular coagulation and acute respiratory distress syndrome at the ages of 22, 29 and 33 years, respectively.

Upon presentation to our department, the patient reported fever and pain in his left lower leg. Physical examination revealed bilateral limb lymphoedema and redness and swelling extending from the left lower leg to the thigh (Fig. 3c), accompanied by exudates (Fig. 3d). Blood tests revealed elevated C-reactive protein and procalcitonin levels, indicating inflammation. Transthoracic echocardiography did not reveal valvular vegetation. Given the previous sepsis episode attributed to *Streptococcus agalactiae* (Group B *Streptococcus* (GBS)), ampicillin treatment (8 g per day) was initiated. Blood culture results for this episode were negative. Owing to improved clinical and laboratory findings, the patient was discharged on day 9.

After discharge, patient 2 was referred for plastic surgery at another hospital, where he underwent vascularized lymph node transplantation. Cellulitis recurred only once postoperatively, but the patient did not require hospitalization. There has been no recurrence for over 3 years (Fig. 3e), indicating successful intervention and significant improvement in his condition. Genetic testing revealed a heterozygous variant, *RIT1* (OMIM: 609591) chr1(GRCh37:g.155874247C>G), NM_006912.6:c.284G>C (p.Gly95Ala), that was previously reported as a pathogenic variant^10^.

### Literature review and combined data analysis

The literature search across PubMed with the MeSH term ‘Noonan syndrome’ combined with the aforementioned text words resulted in 85 hits. Based on the title, abstract and full text, 68 records were excluded because they did not meet the eligibility criteria or were not available in full text, and 8 duplicates were removed. In total, 9 articles (7 case reports and 2 case series) were identified, involving 16 patients with NS complicated by lymphoedema and cellulitis (Fig. 1). The genetic findings and clinical features are summarized in Table 1.

**Table 1:** Summary of clinical information for 18 patients with Noonan syndrome having lymphoedema and cellulitis.

Report, year	Age (years)	Sex	Causative gene	Cardiac anomaly	Lymphedema onset (age, years)	Cellulitis		Bacteremia or sepsis		Treatment with antibacterial agents	Surgical or prophylactic treatment
						First occurrence (age, years)	Number of episodes	Occurrence (age, years)	Causative bacteria		
Minkin W, et al. (1974)^27^	58	M	N/A	N/A	Since early childhood	58	N/A	N/A	N/A	Topical antibiotics	N/A
Miller M, et al. (1978)^28^	27	M	N/A	MVP	16	27	1	N/A	N/A	N/A	N/A
White SW, et al. (1984)^29^	17	M	N/A	N/A	N/A	17	Many	N/A	N/A	N/A	N/A
	14	M	N/A	N/A	N/A	N/A	Several	N/A	N/A	N/A	N/A
	9	F	N/A	N/A	N/A	N/A	Several	N/A	N/A	N/A	N/A
	12	M	N/A	N/A	N/A	12	5	N/A	N/A	N/A	N/A
Fuchs S, et al. (2015)^30^	14	M	N/A	ASD, VSD	14	14	N/A	N/A	N/A	N/A	N/A
Joyce S, et al. (2016)^6^	19	M	*PTPN11*	ASD, PS, HCM	14	N/A	≥2	N/A	N/A	N/A	N/A
	26	M	*PTPN11*	N/A	4	17	1	N/A	N/A	N/A	N/A
	11	M	*PTPN11*	ASD, PS	6	N/A	1	11	*S. pyogenes* (GAS)	N/A	N/A
	63	M	*PTPN11*	N/A	53	52	2	N/A	N/A	N/A	Endovenous laser ablation (for lymphorrhea of the penis and scrotum)
	17	M	*RIT1*	PS	6	N/A	N/A	N/A	N/A	N/A	Skin graft surgery (for scrotal lymphorrhea)
Milosavljević D, et al. (2016)^31^	32	F	*RIT1*	MVP	12	N/A	≥2	N/A	N/A	N/A	Surgical procedures (details unknown)
Suzuki K, et al. (2016)^11^	19	M	N/A	ASD, PS	N/A	19	2	19 19	*SDSE* (GGS) *SDSE* (GGS)	ABPC ABPC	LVA 1 g per day TMP–SMX
Ding Y, et al. (2019)^32^	9	F	*SOS2*	ASD, VSD	3	3	N/A	N/A	N/A	N/A	N/A
Koike T, et al. (2023)^12^	28	M	N/A	Tetralogy of Fallot	25	26	4	28 29	*Citrobacter braakii* *Escherichia coli*	TAZ–PIPC, CTRX SBT–ABPC	LVA Low-dose TMP–SMX
Patient 1	25	M	*RIT1*	ASD, PS, HCM	14	16	≥10	19 21 22 25	*Escherichia coli* *S. pyogenes* (GAS) *Staphylococcus aureus* *SDSE* (GGS)	TAZ–PIPC CTRX, CEZ SBT–ABPC, CEZ SBT–ABPC	LVA
Patient 2	36	M	*RIT1*	MVP, TR	13	13	≥10	31 33 33 35	*Staphylococcus hominis* (GGS) *S. agalactiae* (GBS) *S. agalactiae* (GBS)	N/A N/A N/A ABPC	Lower limb debulking surgery LVA VLNT

*M* male, *F* female, *N/A* not available or applicable, *MVP* mitral valve prolapse, *ASD* atrial septal defect, *VSD* ventricular septal defect, *PS* pulmonary stenosis, *HCM* hypertrophic cardiomyopathy, *TR* tricuspid regurgitation, *S* Streptococcus, *SDSE* Streptococcus dysgalactiae subspecies equisimilis, *GAS* group A streptococcus, *GGS* group G streptococcus, *GBS* group B streptococcus, *TMP–SMX* trimethoprim–sulfamethoxazole, *ABPC* ampicillin, *SBT* sulbactam, *TAZ–PIPC* tazobactam–piperacillin, *CTRX* ceftriaxone, *CEZ* cefazolin, *LVA* lymphovenous anastomosis, *VLNT* vascularized lymph node transplant, Tetralogy of Fallot includes VSD, overriding aorta, PS and right ventricular hypertrophy.

Data of 18 patients (15 men and 3 women) with NS complicated by cellulitis (our hospital’s cases combined with literature reports) were analyzed. Genetic analysis was conducted for eight patients, revealing *PTPN11*, *RIT1* and *SOS2* variants in four, four and one, respectively. The cardiac defects observed included ASD and PS in six patients each, mitral valve prolapse in three, HCM and ventricular septal defect in two, and tricuspid regurgitation in one. Sepsis developed in 5 patients across 13 episodes, with 4 patients having ASD and PS. Transoesophageal echocardiography in these sepsis cases did not reveal vegetation. The average age for lymphoedema onset was 15.0 years, with a median of 13.5 (range, 3–53) years. Cellulitis first occurred at the average and median ages of 22.8 and 17.0 years, respectively (range, 3–58), predominantly in adolescence, except for two cases where it occurred at an older age. Five patients experienced bacteremia, sepsis or septic shock owing to cellulitis in 13 episodes; pathogens identified were GAS in two episodes, GBS in two episodes, group G *Streptococcus* species (GGS, including *Streptococcus dysgalactiae subspecies equisimilis* (SDSE)) in four episodes, *Escherichia coli* in two, and one episode each of *Citrobacter braakii, Staphylococcus aureus* and *Staphylococcus hominis*. Details concerning antibiotics used were available for four patients: two patients from the literature^11,12^ and two of our patients. Penicillin, such as tazobactam–piperacillin or sulbactam–ampicillin, or cephem, such as ceftriaxone or cefazolin, was initiated or deescalated once the blood culture revealed the causative agent. A prophylactic trimethoprim–sulfamethoxazole regimen was postoperatively administered to two patients^11,12^. No recurrence was observed in one patient^11^; however, the other patient experienced three episodes of cellulitis in the following year and was treated with other antibiotics^12^. Surgical interventions for lymphoedema included lymphovenous anastomosis in four patients who subsequently experienced recurrent cellulitis and/or bacteremia. Patient 2 from our case study underwent lower limb debulking surgery with limited efficacy; however, subsequent lymph node transplantation significantly reduced recurrent cellulitis. Additional treatments included skin grafting for lymphorrhoea (lymph or chyle leakage) of the penis and scrotum and intravenous laser ablation in separate cases^6^, highlighting varied and innovative approaches to managing NS complications.

## Discussion

Bilateral lower limb lymphoedema commonly emerges in later childhood or adulthood in patients with NS^6^. Similarly, the patients with NS in our study presented with lymphoedema from an average age of 15.0 years. Cellulitis based on lymphoedema occurred from an average age of 22.8 years, and most patients experienced recurrence. These findings indicate that an early onset of lymphoedema persists and progresses over time, leading to a vicious cycle of cellulitis and lymphoedema. This early manifestation and the recurrence pattern emphasize the need for vigilant monitoring and proactive and comprehensive intervention from a young age. Moreover, we explored the genetic and clinical features in the subgroup of patients with NS complicated by lymphoedema and recurrent cellulitis to develop targeted management strategies to mitigate the impact of these complications. Our study showed a male predominance in lymphoedema and recurrent cellulitis cases within the NS population, diverging from the typically reported lack of sex-specific distribution in broader NS demographics^13^.

Pathogenic variants of *PTPN11*, *SOS1*, *RIT1* and *KRAS* have been identified in NS at approximately 40%, 11%, 5% and 2.5%, respectively^14^. Among the nine cases in our cohort, in which genetic analysis was performed, pathogenic variants of *PTPN11*, *SOS2* and *RIT1* were identified in 44.4% (*n* = 4), 11.1% (*n* = 1) and 44.4% (*n* = 4) of patients, respectively (Table 1). This distribution showed a particularly higher prevalence of the *RIT1* variant in our study than the corresponding in previous studies. Although constrained by our small sample size, the prevalence of *RIT1* variants among patients experiencing recurrent cellulitis may indicate a potential genotype–phenotype correlation, suggesting that specific genetic backgrounds could predispose individuals to certain clinical manifestations, including the development of lymphoedema and recurrence of cellulitis.

Congenital heart disease is one of the most important problems in the long-term follow-up of NS, affecting over 67.4–80% of the patients^15,16^. The predominant cardiac conditions in NS include PS, HCM and ASD, with prevalence rates of 25–35%, 20% and 6–10%, respectively^9,14^. In our cohort, the frequencies of PS and HCM aligned with these established rates at 33.3% (*n* = 6) and 11.1% (*n* = 2), respectively. However, the prevalence of ASD was higher (33.3%, *n* = 6) in our study than that observed previously (Table 1). In addition, the development of sepsis following cellulitis was observed in patients with NS having PS and ASD (*n* = 4, 80%, respectively), suggesting the impact of specific cardiac anomalies on the risk and severity of infections in this patient population.

Ichikawa et al. reported the prevalence of PS, HCM and ASD among individuals with *PTPN11* variants at 32%, 20% and 32%, respectively (*n* = 25), and among individuals with *RIT1* variants at 80%, 20% and 40%, respectively (*n* = 5)^15^. We more frequently identified ASD (*n* = 3, 75%) with *PTPN11* variants and variable cardiac conditions, including mitral valve prolapse (*n* = 2, 50%) and tricuspid regurgitation (*n* = 1, 25%), with *RIT1* variants (Table 2). These findings suggest that the pathogenic variants also affect the phenotype developing lymphatic abnormalities of susceptibility to cellulitis, in addition to the incidence of cardiac anomalies.

**Table 2:** Prevalence of cardiac anomalies with pathogenic *PTPN11* and *RIT1* variants in Noonan syndrome.

	*PTPN11* variant		*RIT1* variant	
	Patients with NS (*n* = 25) reported by Ichikawa, et al.^15^	Patients with NS complicated by lymphoedema and cellulitis (*n* = 4) (our cohort)	Patients with NS (*n* = 5) reported by Ichikawa, et al.^15^	Patients with NS complicated by lymphoedema and cellulitis (*n* = 4) (our cohort)
PS	8/25 (32%)	2/4 (50%)	4/5 (80%)	2/4 (50%)
HCM	5/25 (20%)	1/4 (25%)	1/5 (20%)	1/4 (25%)
ASD	8/25 (32%)	3/4 (75%)	2/5 (40%)	1/4 (25%)
MVP	N/A	0/4 (0%)	N/A	2/4 (50%)
TR	N/A	0/4 (0%)	N/A	1/4 (25%)

*NS* Noonan syndrome, *PS* pulmonary stenosis, *HCM* hypertrophic cardiomyopathy, *ASD* atrial septal defect, *MVP* mitral valve prolapse, *TR* tricuspid regurgitation, *N/A* not available or applicable.

In general, *Streptococcus* species, along with *Staphylococcus* species, are major cellulitis pathogens^17^. Among these, GAS, GBS and GGS contribute to 34.6%, 26.9% and 10.8% of streptococcal bacteremia cases, respectively^18^. SDSE, one of the GGS, has been increasingly isolated from patients with various infection, including skin and soft tissue infections. It easily invades the deep tissues and bloodstream^19^, persists in the tissues and results in recurrence even after antibiotic therapies^11,20^. SDSE bacteremia occurs most commonly in older patients; however, it also poses a risk to younger individuals with predisposing factors, especially lymphatic disorders. Moreover, the rate of recurrence of GGS bacteremia has been reported to be 5–10%, higher than that of GAS bacteraemia^11^. We recorded 13 episodes of bacteremia or sepsis among five patients, with GAS, GBS and GGS emerging as pathogens in 23.1%, 23.1% and 30.8% of cases, respectively. In our study, one patient with NS had experienced recurrent SDSE bacteremia^11^. Although no major immune disturbance was found with patients with NS in a previous study^21^, our finding suggests that they possess GGS infection vulnerabilities, irrespective of their relatively younger age.

Empirical cellulitis treatment typically involves semi-synthetic penicillins, first- or second-generation cephalosporins, macrolides or clindamycin^22^. Details concerning antibiotics used were available for four patients in our study. Penicillin or cephem was initiated or deescalated once the blood culture revealed the causative agent, which proved effective in all four cases, achieving immediate improvement. However, all four patients had experienced recurrence of cellulitis even after antibiotic therapy. This finding suggests that the amount and duration of the antibiotics were not sufficient for clearing the persistent bacteria.

Despite limited evidence supporting their long-term use, prophylactic antibiotics have become common in preventing cellulitis recurrence^23^. Trimethoprim–sulfamethoxazole is frequently administered for long terms owing to its mild adverse effect profile^24^. In our study, the prophylactic trimethoprim–sulfamethoxazole regimen was postoperatively administered to two patients^11,12^. No recurrence was observed in one patient with SDSE bacteremia^11^; however, the other patient with *Citrobacter braakii* bacteraemia experienced three episodes of recurrent cellulitis in the following year after starting trimethoprim–sulfamethoxazole. Our findings suggest that prophylactic antibiotic monotherapy is not an ideal strategy for preventing recurrence.

Strategies to mitigate recurrent cellulitis include addressing interdigital maceration; maintaining skin hydration with emollients to prevent dryness and cracking; and reducing underlying lymphoedema through limb elevation, compression therapy and potentially diuretic therapy^22^. Compression therapy is crucial and involves using elastic stockings and manual lymphatic drainage, and surgical interventions are considered along with these conservative treatments. Surgical options, including lymphovenous anastomosis or bypass, vascularized lymph node transplant and debulking procedures including liposuction or direct excisional procedures, have been promising^25^. Lymphovenous anastomosis aims to bypass obstructed lymphatic vessels to adjacent venules for early-stage lymphoedema, whereas vascularized lymph node transplant, a newer technique for more advanced cases, involves transplanting lymph nodes to the affected area, facilitating lymphangiogenesis and improving lymphaticovenous drainage, driven by perfusion gradients between the arterial inflow and venous outflow^25^. In our cohort, although lymphovenous anastomosis was performed in four patients, postoperative recurrence was observed in all cases. By contrast, vascularized lymph node transplant in patient 2 caused no recurrence, implying its effectiveness in patients with a history of recurrent cellulitis and severe complications, such as disseminated intravascular coagulation and acute respiratory distress syndrome. Recently, the potential use of Ras/mitogen-activated protein kinase inhibitors, such as trametinib, has shown efficacy in partially reversing NS-associated lymphatic hyperplasia^26^. These innovative treatments offer hope for lymphoedema management in patients with NS.

There are some limitations to the scope of our literature review and the nature of the data collected. This diversity, along with potential publication bias, may complicate the generalizability of our findings. The scarcity of case reports and series further limited our access to comprehensive data on genetic testing and clinical features, making it challenging to delineate detailed genotype–phenotype correlations. Furthermore, the small sample size introduces the risk of over- or underestimating the findings. This report, focusing on recurrent cellulitis in patients with NS and summarizing 18 cases, highlights the emergence of lymphoedema in adolescence, which predisposes patients to subsequent cellulitis and sepsis episodes. Despite the limitations posed by the small size of our cohort, these findings underscore the need for early lymphoedema intervention to mitigate the risk of recurrent cellulitis and its potential progression to life-threatening conditions.

In conclusion, effective management strategies should include the judicious use of antibiotics for acute treatment and prevention, along with a combination of conservative measures and surgical interventions toward individual patient needs. Furthermore, the promising role of novel therapeutic agents, including Ras/mitogen-activated protein kinase inhibitors, in treating NS-associated lymphoedema warrants further investigation. Our findings highlight the critical need for studies to refine treatment approaches and enhance outcomes in patients with these complex genetic disorders.

## Data Availability

The datasets used and/or analyzed during the current study are available from the corresponding author upon reasonable request.
